# Neuroanatomy of the spinosaurid *Irritator challengeri* (Dinosauria: Theropoda) indicates potential adaptations for piscivory

**DOI:** 10.1038/s41598-020-66261-w

**Published:** 2020-06-09

**Authors:** Marco Schade, Oliver W. M. Rauhut, Serjoscha W. Evers

**Affiliations:** 1 Institute of Geography and Geology, Palaeontology and Historical Geology, University of Greifswald, 17489 Greifswald, Germany; 2 Department of Earth and Environmental Sciences, Palaeontology and Geobiology, Ludwig-Maximilians-Universität, 80333 München, Germany; 3 Bayerische Staatssammlung für Paläntologie und Geologie, Staatliche Naturwissenschaftliche Sammlungen Bayerns (SNSB), 80333 München, Germany; 4 GeoBioCenter, Ludwig-Maximilians-Universität, 80333 München, Germany; 5 Department of Geosciences, University of Fribourg, 17000 Fribourg, Switzerland

**Keywords:** Neuroscience, Evolution, Palaeontology

## Abstract

Spinosauridae, a theropod group characterized by elongated snouts, conical teeth, enlarged forelimbs, and often elongated neural spines, show evidence for semiaquatic adaptations and piscivory. It is currently debated if these animals represent terrestrial carnivores with adaptations for a piscivorous diet, or if they largely lived and foraged in aquatic habitats. The holotype of *Irritator challengeri*, a nearly complete skull from the late Early Cretaceous Santana Formation of northeastern Brazil, includes one of the few preserved spinosaurid braincases and can provide insights into neuroanatomical structures that might be expected to reflect ecological affinities. We generated digital models of the neuroanatomical cavities within the braincase, using computer tomography (CT) data. The cranial endocast of *Irritator* is generally similar to that of other non-maniraptoriform theropods, with weakly developed distinctions of hindbrain and midbrain features, relatively pronounced cranial flexures and relatively long olfactory tracts. The endosseous labyrinth has a long anterior semicircular canal, a posteriorly inclined common crus and a very large floccular recess fills the area between the semicircular canals. These features indicate that *Irritator* had the ability for fast and well-controlled pitch-down head movements. The skull table and lateral semicircular canal plane are strongly angled to one another, suggesting a downward angling of approximately 45° of the snout, which reduces interference of the snout with the field of vision of *Irritator*. These neuroanatomical features are consistent with fast, downward snatching movements in the act of predation, such as are needed for piscivory.

## Introduction

Spinosauridae is a large-bodied theropod group within Megalosauroidea known from the Cretaceous, although their phylogenetic relationships indicate that the clade must have originated in the Jurassic^1^. Spinosaurids are characterized by a long and slender skull, conical teeth, strongly developed forelimbs with exceptionally large thumb claws and elongated neural spines^2–6^. Due to superficial similarities in cranial form with piscivorous Crocodilia, such as the gharial, and the wealth of fossil fish within the assemblages they were found in, spinosaurids were repeatedly associated with a semiaquatic lifestyle and piscivory [e.g.^3,7–12^]. Direct evidence for piscivory comes from acid-etched fish scales in the stomach contents of *Baryonyx walkeri*^3^, although the same individual also includes terrestrial dinosaur bones of a juvenile ornithopod. Predation on pterosaurs has also been shown for spinosaurids^13^. Thus, direct evidence for spinosaurid diets indicates a mix, or opportunistic behaviour with a tendency towards relatively small prey items. Additional evidence to support semiaquatic adaptations beyond dietary preference in spinosaurids comes from: isotope signals acquired from tooth enamel of samples from different geographical contexts, which show that spinosaurids spent a significant amount of their lifetime in water^8,14,15^; the suspected elevated position of the orbits in the skull^12^; the occurrence of pachyostosis in the femur of a specimen referred to *Spinosaurus aegyptiacus*^10^; though see^16^ for taxonomic identification]; and the presence of a fluke-like tail that was probably used for aquatic, tail-propelled locomotion in the same specimen of *S. aegyptiacus*^17^.

**Figure 1:**
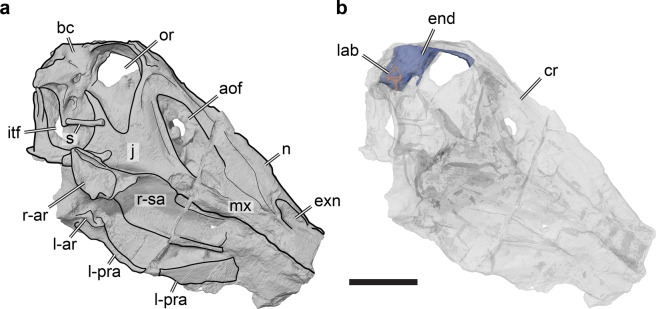
3D renderings of the holotype fossil of *Irritator challengeri* (SMNS 58022) in right lateral view. (**a**) solid rendering of the skull with interpretative line drawing indicating gross anatomy; (**b**) transparent rendering of the skull with solid rendering of the cranial endocast and endosseous labyrinth. Note that the skull is inclined according to the ‘alert’ head pos-ture inferred by lateral semicircular canal horizontality (see text for details). Scale bar equals 100 mm. Abbreviations: aof, antorbital fenestra; ar, articular; bc, brain-case; cr, cranium; end, cranial endocast; exn, external naris; itf, infratemporal fenestra; j, jugal; l-, indicates left element; lab, endosseous labyrinth; mx, maxilla; n, nasal; or, orbit; pra, prearticular; r-, indicates right element; s, stapes; sa, surangular.

Spinosaurid material beyond isolated teeth is rare, making partial skeletons and especially skull remains particularly valuable to test for the presence of ecological adaptations. So far, the only spinosaurid taxon for which an almost complete skull is known is *Irritator challengeri* (SMNS 58022; Staatliches Museum für Naturkunde Stuttgart, Stuttgart, Germany) from the Aptian–Albian Santana Formation of Brazil^18,19^ (Fig. 1). Modern methodological advances, such as computer tomography (CT) scanning methods, can reveal new details of specimens, which in turn give insights into unknown aspects of spinosaurid functional anatomy, ecology and evolution.

The braincase of vertebrates houses the brain and is closely associated with essential sensory organs [see for theropods e.g.^20–26^]. In addition, the braincase provides muscle attachment sites for the jaw and neck muscles, and directly articulates via the first neck vertebra with the postcranial skeleton. Equipped with bony crests as display devices, braincases can even be important for assessing behavioral questions^24^.

In this study, we use CT scanning of the well-preserved braincase *of Irritator challengeri* to reveal its neuroanatomy. *I. challengeri* may represent the apex predator of the Santana Formation, which is one of the most important Early Cretaceous (Aptian–Albian) fossil lagerstätten from South America. The outer morphology of its braincase will be described elsewhere. The digital reconstructions of the endocast and inner ear give new insights into neuroanatomical features and associated sensory organs of this animal, and enable ecological implications to be hypothesized.

## Results

### Cranial endocast and innervation

**Figure 2:**
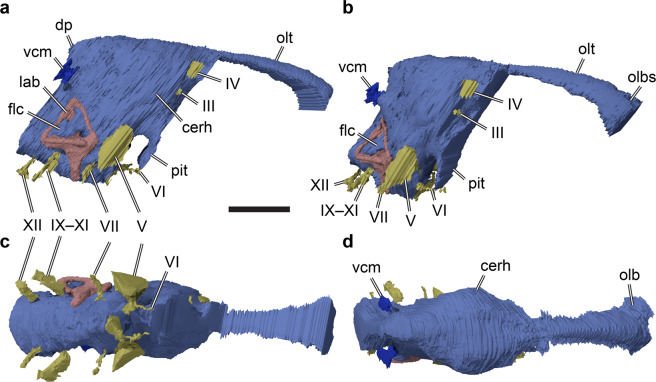
3D rendering of the cranial endocast, cranial nerves, endosseous labyrinth, and as-sociated structures of the holotype fossil of *Irritator challengeri* (SMNS 58022). (**a**) right lat-eral view; (**b**) oblique view, roughly anterolaterally oriented; (**c**) ventral view; (**d**) dorsal view. Scale bar equals 30 mm. Abbreviations: cerh, cerebral hemisphere; dp, dural peak; flc, floc-cular recess; III, oculomotor nerve; IV, trochlear nerve; IX–XI, endocast of the metotic fissure, holding the glossopharyngeal (IX), vagus (X), and accessory (XI) nerves; lab, endosseous lab-yrinth; olb, olfactory bulb; olbs, olfactory bulb sulcus; olt, olfactory tract; pit, pituitary; V, trigeminal nerve; VI, abducens nerve; VII, facial nerve; vcm, dorsal middle cerebral vein; XII, hypoglossal nerve.

The cranial endocast of SMNS 58022 is generally similar to that of other non-maniraptoriform theropods, in that many features of the hindbrain and midbrain (e.g. cerebellum and optic lobes) are not confidently perceivable as distinct structures on the surface of the endocast (Fig. 2). This indicates a poor direct correspondence between neural tissues and endocranial cavity surface, as in many other reptiles including crocodiles, lepidosaurs, and turtles [e.g.^27–30^]. A much closer brain-braincase correspondence is realized in strongly encephalised groups, which include some coelurosaurs, avian theropods, pterosaurs, or mammaliforms [e.g.^31–37^]. The endocast of SMNS 58022 is less tubular than that of crocodiles or many non-avialan coelurosaurs [e.g.^24,29^]. Instead, pontine and cephalic flexures are more pronounced, resulting in a midbrain section of the endocast that is relatively strongly angled between the hindbrain and forebrain (Fig. 2a). This is consistent with observations for basal tetanurans and ceratosaurs [e.g.^20,23,25,38,39^]. Near the cephalic flexure, the endocast of SMNS 58022 shows a weakly developed dural peak (Fig. 2a). However, because of a damage on the parietal, an accurate reconstruction of the area in which the pineal gland would be expected cannot be provided. As in other basal tetanurans and ceratosaurs [e.g.^20,23^], but unlike coelurosaurs^24^, the dorsal middle cerebral vein exits the cranial endocast well below the level of the dural peak (Fig. 2a,b) in SMNS 58022.

In the forebrain, the cerebral hemispheres are distinguishable as laterally expanded but weakly delimited bulbs on the dorsolateral surface of the endocast of SMNS 58022 (Fig. 2). The impressions of the olfactory tracts are preserved along the ventral surface of the frontals. The conjoined impressions of the olfactory tracts and bulbs are around 55 mm in length. Anteriorly, the olfactory tract gets wider and diverges into distinct olfactory bulbs, which are dorsally separated by a shallow sulcus (Fig. 2b,d). Long olfactory tracts are considered plesiomorphic within theropods^24^, and are shortened in theropods closer to the avian crown than basal tetanurans^24^. The full extent of the olfactory bulbs could not be reconstructed for SMNS 58022, due to insufficient preservation anteriorly. Ventral to the base of the olfactory tracts, the orbitosphenoid captures the courses of the cranial nerves III (oculomotor nerve) and IV (trochlear nerve) (Fig. 2a,b). Anteroventrally in the forebrain, the impression of the pituitary fossa is clearly visible in SMNS 58022 (Fig. 2a,b). Although no clear cerebral carotid canal could be identified, the paired abducens nerve (CN VI) canals could be reconstructed (Fig. 2a–c). The position of the foramen for the trigeminal nerve (CN V) is posterodorsal to the abducens canal. The respective foramen is large and clearly visible externally^19^. The facial nerve (CN VII) originates anteriorly to the position of the cochlear duct, whereas the metotic foramen and recessus scalae tympani for CN IX–XI is found posterior to the cochlear duct (Fig. 2a–c). Hypoglossal nerve (CN XII) canals were identified on both sides, whereas Sues *et al*.^19^ were only able to locate one such foramen externally, on the left side of the specimen.

**Figure 3:**
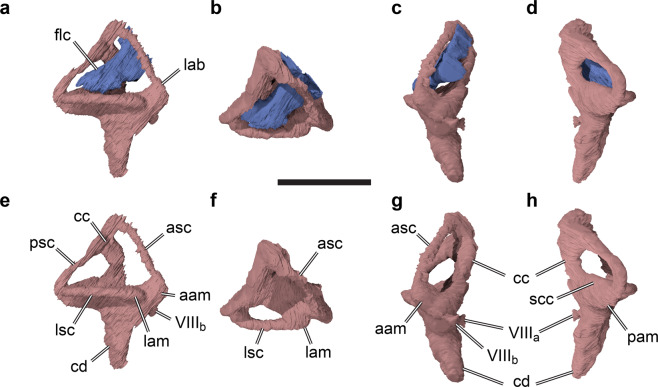
3D rendering of right endosseous labyrinth and floccular recess of the holotype fossil of *Irritator challengeri* (SMNS 58022). (**a**)–(**d**), 3D renderings including the floccular re-cess; (**e**)–(**f**), excluding floccular recess. (**a**), (**e**), lateral view; (**b**), (**f**), dorsal view; (**c**), (**g**), anterior view; (**d**), (**h**), posterior view. Scale bar equals 20 mm. Abbreviations: aam, anterior ampulla; asc, anterior semicircular canal; cc, common crus; cd, cochlear duct; flc, floccular recess; lab, en-dosseous labyrinth; lam, lateral ampulla; lsc, lateral semicircular canal; pam, posterior am-pulla; psc, posterior semicircular canal; scc, secondary common crus; VIIIa, medial branch of vestibulocochlear nerve; VIIIb, anterior branch of vestibulocochlear nerve.

In the midbrain region, somewhat posteroventrally to the cerebral expansion, the endocast shows a posteroventrally directed flap that projects off the cerebellum, the floccular lobe (flocculus in the following; Figs. 2a,b, 3a–d). The flocculus of SMNS 58022 is very large; it projects posteriorly into the space confined by the posterior semicircular canal and secondary common crus of the endosseous labyrinth, and extends laterally to the level of the lateral semicircular canal. Thus, the flocculus of *Irritator challengeri* is much larger than that of other basal tetanurans [e.g.^20,22,25^], and even most coelurosaurs [e.g.^40^], but is similar in size to taxa that reportedly have large flocculi (e.g. *Conchoraptor gracilis*^26^).

The medulla oblongata in the hindbrain is relatively broad mediolaterally, and connected with the foramen magnum.

The volume of the endocranial cavity was measured to be approximately 80 cm^3^ (measured as suggested in^23^).

### Endosseous labyrinth

The endosseous labyrinth of SMNS 58022 is composed of the dorsally positioned vestibular system that includes the semicircular canals, and a ventrally tapering cochlear duct (Fig. 3). The cochlear duct is relatively long and dorsoventrally as tall as the semicircular canal system (Fig. 3a,e). It is gently ventromedially inclined (Fig. 3c,d,g,h). The vertical semicircular canals are strongly asymmetrical: the common crus is posteriorly directed rather than strictly dorsally (Fig. 3e). As a consequence, the posterior semicircular canal forms a low and relatively short arc, whereas the anterior semicircular canal is long and posterodorsally forms a 180° turn to reach the common crus. The course of the posterior semicircular canal does not lie in a single vertical plane, but the midpart of the canal is slightly bowed anterolaterally. In many tetrapods, the posterior portion of the lateral semicircular canal and the ventral portion of the posterior semicircular canal intersect, and form a singular cavity, the secondary common crus^30^. In SMNS 58022, the posterior and lateral semicircular canal also intersect, but the courses of the individual paths of the membranous ducts within the intersection are still clearly visible in the endosseous labyrinth model as impressions within the secondary common crus. Although these impressions are difficult to see in standard orientation figures of the endosseous labyrinth model (Fig. 3d,h), they are clearly visible in the actual 3D model (see^41^). The posterior semicircular canal arcs ventrally underneath the lateral semicircular canal, which curves medial to the posterior canal toward the common crus. The imprints of the posterior LSC and ventral PSC portions provide evidence that the semicircular ducts were relatively widely separated in life, as is the case in many modern birds, in which a secondary common crus is largely absent^42^.

### Pneumatic cavities

Pneumatic cavities in the braincase of SMNS 58022 are present, but hard to delimitate within our CT data. This is in part because some pneumatic cavities, particularly the caudal tympanic recess within the paroccipital process, and a recess directly ventral to the brain within the basioccipital, tentatively identified as the medial subcondylar recess [see ^24,43^ for theropod braincase pneumaticity], are interrupted by extensive webbing of laminae. Seemingly, there are more pneumatic recesses present within the braincase of SMNS 58022, including minor cavities within the basioccipital and the prootic, as well as a basisphenoid recess and a subsellar recess within the basisphenoid. In very general terms, the extent of braincase pneumaticity seems to be higher than in ceratosaurs^23,44^, but less than in tyrannosaurs^24^.

## Discussion

### Auditory capabilities

Auditory capability and cochlear duct length have been hypothesized to be correlated among extant archosaurs^45^. We used the equations derived by Walsh *et al*.^45^ alongside digital measurements (cochlear duct length = 18.1 mm; basicranium length = 75.3 mm) to infer the mean hearing frequency (1950 Hz) and frequency band width (3196 Hz) for *Irritator challengeri*. We only consider these values as rough guidance, but the resulting frequency range between c. 350–3550 Hz places *I. challengeri* around the lower end of the sensitivity range inferred for modern birds, but above that for crocodiles^45^. Furthermore, the estimates are in approximate agreement with those calculated for other theropod dinosaurs [e.g.^40^]. Additional cues for the auditory capabilities can possibly be inferred from pneumaticity. Increased volume achieved by extensive tympanic pneumaticity, as observed in extant Aves and Crocodilia, but also tyrannosaurs and many maniraptoran theropods^46^, is thought to impact impedance matching of the middle ear by reducing the acoustic stiffness and improving stapes vibration. These effects facilitate the reception of low frequencies, as well as the amplification of frequency-depending sounds^24,29,47^. The lesser degree of tympanic pneumatization of SMNS 58022, which probably represents a symplesiomorphy shared with other basal tetanurans, might indicate that the middle ear of *I. challengeri* was less specialized than those of tyrannosaurs. It is noteworthy that the stapes of SMNS 58022 is relatively more robust than in some other basal tetanurans, such as *Allosaurus* spp.^48,49^, which might negatively affect the efficiency of the acoustic transformer ratio, and thus of impedance matching, of *I. challengeri*.

### Vestibular anatomy as a guide to ecological reconstructions?

It is currently unclear how informative labyrinth geometry is for inferring habitat ecology in reptiles. The semicircular canals, which are the focus of most studies that test for such correlations, are used in gaze stabilization by detecting angular accelerations of the head as inputs to the vestibulo-ocular (VOR) and vestibulo-collic (VCR) reflexes^50^. Endolymphatic flow within the inner ear organ is determined by semicircular canal geometry, and the shape of the vestibular organ is therefore expected to vary depending on locomotor mode, as has been found for many mammal groups [e.g.^51–54^]. Functional changes in vestibular anatomy are expected to be largest in groups that experienced strong ecological transitions, such as the evolution of secondarily marine lifestyles or flight. However, for both these transitions, no characteristic shape change uniquely linked to either ecological adaptation has yet been found. In birds, studies that test for ecological signals in the vestibular anatomy so far fail to find correlations with flight ability or style. For instance, the size of the floccular lobe does not indicate flight ability^35^. Benson *et al*.^42^ found little locomotor signal within the semicircular canal system size or semicircular canal shape of birds. They suggest that other constraints, such as visual acuity, head size, and spatial constraints within the cranium could instead determine the major variation associated with labyrinth shape^42^. Some studies have reported potential aquatic adaptations to the endosseous labyrinth in non-dinosaurian reptiles^30,55–57^. However, the hypothesis that aquatic tetrapods have low aspect ratios (i.e. comparatively dorsoventrally low and anteroposteriorly long labyrinths^55^;) is not supported by more recently collected data^30^. Additionally, thick endosseous semicircular canals, although found in several secondarily marine groups^30,56,57^, can also be present in highly terrestrial animals^30^. Furthermore, the endosseous labyrinths of phylogenetically shallow marine lineages, such as penguins or seals seem to be influenced more strongly by other factors like phylogeny (penguins:^55,56^; neodiapsids:^58^), or neck agility (seals:^59^), rather than habitat ecology.

Despite the above-mentioned reservations against clear ecological signals in the vestibular anatomy of reptiles, potential functional adaptations of the dinosaurian labyrinth have been reported throughout the literature. For instance, changes in labyrinth geometry associated with the evolution of bipedality within dinosaurs, particularly the elongation of the vertical semicircular canals, have been recorded for dinosaurs^60^. On the other hand, these changes could not be found in an ontogenetic labyrinth series of the dinosaur *Massospondylus carinatus*^61^, which experiences a change from quadrupedality to bipedality during ontogeny^62^.

Spinosaurids are deeply nested within the Theropoda, a clade of obligate bipedal, comparatively agile, and terrestrial animals. Even if spinosaurids had a semiaquatic ecology [e.g.^10^], it is quite possible that ancestral constraints on the theropodan bauplan dominate the shape of the spinosaurid labyrinth [e.g.^58^]. Therefore, semiaquatic adaptations are not necessarily expected to be overly obvious, or present at all, and their absence cannot be taken as strong evidence against semiaquatic lifestyles. In the following, we discuss and interpret three aspects of the neuroanatomy of *Irritator challengeri* that have been the focus of many studies that try to synthesize ecological adaptations from neuroanatomical structures: the size of the floccular recess, the size of the anterior semicircular canal, and the relative orientation of the lateral semicircular canal.

### Behavioral interpretations of neuroanatomy

SMNS 58022 shows enlarged floccular recesses. The flocculus is important in the control and coordination of head, eye, and neck movements during gaze stabilization, by being involved in processing the vestibulo-ocular (VOR) and vestibulo-collic (VCR) reflexes^33,35,51^. Additionally, the flocculus plays a role in the reflex control of neck movements^63^. Although the floccular recess may also house non-neural tissues^35^, floccular size has been interpreted to be grossly indicative of the amount of neural tissue and, by inference, the amount of respective signal procession^33^. A small flocculus endocast is conversely not necessarily indicative of a small amount of respective neural tissue, because the floccular lobe also extends within the cerebellum of extant birds^29,35^. However, a reduction in floccular size in the abelisaurid certosaurian *Majungasaurus crenatissimus* has been interpreted to indicate a decreased reliance on quick movement and sophisticated gaze-stabilization mechanisms in this taxon^23^, though not necessarily in other abelisaurids^44,64^. In pterosaurs, enlarged floccular recesses have been interpreted as an adaptation to eye-guided pursuit hunting of fish, albeit aerially^33^. Within non-avian theropod dinosaurs, large floccular recesses are common among coelurosaurs, but this structure seems relatively smaller in basal tetanurans [e.g.^20,26,40,44^]. Among birds, flocculus sizes vary. It is noteworthy that particularly large flocculus sizes have been noted for many waterbirds (Procellariiformes, i.e. albatrosses and kin; Phaethoniformes, i.e. tropicbirds; Charadriiformes, i.e. gulls and kind; Anseriformes, i.e. ducks, geese, and swans; Gaviiformes, i.e. loons) and birds with particularly long necks (Rheiformes, i.e. Rhea; Ciconiiformes, i.e. storks)^35,64^. However, Falconiformes (falcons) and some Passeriformes (perching birds) also have large floccular sizes^35^. Although interpretations of floccular sizes are not straightforward [e.g.^35^], we interpret the large floccular size of *Irritator challengeri* as indicative for the relative importance of VOR and VCR coordination, particularly because large floccular sizes are unusual for the inferred phylogenetic position of spinosaurids.

Additional possible behavioural clues come from the semicircular canal system, particularly the anterior and lateral semicircular canals. In *Irritator challengeri*, the anterior semicircular canal is particularly long, with its length being furthermore increased by the posterodorsally inclined common crus. As the anterior semicircular canal is more sensitive to pitch-down movements of the head than the other semicircular canals^65,66^, we suggest that this kind of sensitivity was particularly important for *I. challengeri*. Research suggests that ‘alert’ head orientation can be inferred for animals by aligning the plane of the lateral semicircular canal with a horizontal plane paralleling the ground^33,50,66–68^. Arranging the lateral semicircular canal in SMNS 58022 horizontally, its skull is inclined downwards at approximately 45° (Fig. 1b). This represents a rather strong ventral orientation of the snout tip, and could be interpreted to maximize the field of binocular vision by avoiding obstruction by the elongated snout [see also ^24^]. This approach of inferring head posture has been criticized^69^ based on data that show that modern birds have a range of realized orientation values of the lateral semicircular canal that deviate from horizontality by up to c. 20° to either side of the horizontal plane^67^. However, the same data show that birds with long beaks, such as storks, tend to have labyrinths that are pitched downward with their lateral semicircular canal planes^67,69^, which would even increase the downward inclination inferred for *I. challengeri*. Even extremely upward pitched labyrinth orientations of 20° would result in a strong ventral inclination of the head of *I. challengeri* by approximately 25°. Furthermore, strong downward orientation of the snout of *I. challengeri* is independently supported by the slightly posteroventrally rotated occipital condyle. Therefore, we think that a strong downward orientation of approximately 45° is supported for *I. challengeri* despite the caution that is warranted when making inferences about head orientation^69^.

The above features – particularly a good eye-head coordination, sensitivity for pitch-down movements, and a ventrally inclined snout facilitating three-dimensional vision – are features that are presumably important for pursuit hunters, particularly for animals that hunt prey that is small and agile in comparison to their own body size. These findings are consistent with data from skull mechanics and functional anatomy in spinosaurids^7,70^. Although the known direct evidence for predation in spinosaurids^3,13^ indicates an opportunistic feeding behaviour, fish might have played an important role in the diet of these animals. The ‘alert’ head posture of *Irritator challengeri*, in which the snout is held downward, furthermore possibly allowed the animal to tuck its snout into the water, while the retracted nares and eyes were not submerged. Possible mechanoreceptor foramina similar to those of crocodiles^71^ have been identified for spinosaurids^10^, although such structures can also be found in clearly terrestrial theropods^72^. The enlarged anterior semicircular canal facilitated fast downward movements, which were coordinated in part by a large floccular recess, and in conjunction allowed snapping movements during hunting of small prey. This functional hypothesis requires fast neck movements. Thus, independent skeletal evidence in support of our interpretation might come from the morphology of the cervical vertebrae of spinosaurids. Although this part of the skeleton is not preserved in *I. challengeri*, certain features of spinosaurid cervical morphology are consistently present among different members of the group, justifying the use of comparative evidence^1,16^. Spinosaurids have comparatively long anterior and mid-cervical centra^16^, which make their necks considerably longer than those of other large-bodied theropods. Strong ventral rugosities on the mid-cervical to posterior centra of the spinosaurid *Sigilmassasaurus brevicollis* have been interpreted as osteological correlates for strong dorsoventral flexion musculature^16^. All of this evidence indicates a specialization of these gigantic predators on considerably smaller and elusive prey, including fish, which is remarkably different from that of other large-sized theropods, such as tyrannosaurids or carcharodontodsaurids [e.g.^73–75^], providing further evidence for niche partitioning between coeval spinosaurid and non-spinosaurid theropod taxa.

## Conclusions

*Irritator challengeri*, the first spinosaurid for which neuroantomical features are documented, has a cranial endocast that shows features consistent with the inferred phylogenetic position of spinosaurids as basal tetanurans. These include weakly demarcated brain regions, elongate olfactory tracts and pronounced cranial flexures. *I. challengeri* has an enlarged floccular recess, which is an unusual feature for basal tetanurans. The vestibular part of the endosseous labyrinth is characterized by a large anterior semicircular canal. A large flocculus and anterior semicircular canal indicate that *I. challengeri* could move its head downwards in a fast and coordinated fashion. The lateral semicircular canal orientation suggests a downward inclined snout posture, which enables unobstructed, stereoscopic forward vision, important for distance perception and thus precise snatching movements of the snout. The suite of neuroanatomically facilitated behavioural capabilities inferred for *I. challangeri* are those expected for animals that mostly hunt small and agile prey. Although these prey items could be small terrestrial animals, our interpretations are consistent with, and corroborate independent evidence for the hypothesis for an at least partially piscivorous diet of spinosaurids.

## Materials and Methods

The holotype and only known specimen of *Irritator challengeri* (SMNS 58022) is an almost complete skull (Fig. 1). It is about 55 cm long and well preserved, lacking only the premaxillae and a few other skull bones, especially of the splanchocranium. The specimen has suffered from slight transverse compression and disarticulation of the posterolateral parts of the skull roof, although some of the disarticulated elements are preserved in displaced positions within the skull, such as the postorbital^19^.

We scanned SMNS 58022 originally with a medical Siemens Somatom Force CT scanner (dual source) (voltage: 120 kV, X-ray tube current: 1365 μA, exposure time: 154 ms, voxel size: 0.703123 mm ×0.703124 mm ×3 mm) in the German Heart Centre in Munich. This scan was the base for all digital reconstructions shown herein, except the inner ear and flocculus, which were visible but poorly resolved in the original scan. In order to get higher resolution data for the labyrinth reconstruction, we conducted a second scan focused only on the braincase, using a Zeiss Metrotom 1500 (voltage: 180 kV, X-ray tube current: 1800 μA, exposure time: 250 ms, voxel size: 0.09713 mm) in a subsidiary of Zeiss in Essingen. Digital segmentation and measurements were produced with Amira (5.6.). We used manual segmentation to create our models. Although the density contrast between the cranium and sediment infill of internal spaces was relatively weak, the boundary between bone surface and sediment infill is clearly visible in the slice data. 3D models of the high-resolution flocculus and endosseous labyrinth were aligned with the respective low-resolution structures in Blender 2.79b to get composite figures of models from both scans. A composite neuroanatomical model, as well as individual 3D models and the two CT scans are deposited online^41^.

## Data Availability

The CT slice data and 3D files of SMNS 58022, are published online^41^, in the repository MorphoSource, Project P 951: https://www.morphosource.org/Detail/ProjectDetail/Show/project_id/951.
